# Meteorological Tables for the Month of July, at San Francisco, Sacramento, Marysville, Mormon Island, and Stockton

**Published:** 1855-05

**Authors:** Theodore S. McLellan

**Affiliations:** Brunswick, Me. San Francisco


ART. III.—*Meteorological Tables for the Month of July at San Francisco, Sacramento, Marysville, Mormon Island, and Stockton.* By THEODORE S. McLELLAN, Brunswick, M.

| SAN FRANCISCO. | | | | | | | SACRAMENTO. | | | | MARYSVILLE. | | | | MORMON ISLAND. | | | | STOCKTON. | | | |
|---|---|---|---|---|---|---|---|---|---|---|---|---|---|---|---|---|---|---|---|---|---|---|
| THERMOMETER. | | | | BAROMETER. | | | THERMOMETER. | | | | THERMOMETR. | | | | THERMOMETER. | | | | THERMOMETER. | | | |
| July. | Morn. | Noon. | Even. | Morn. | Noon. | Even. | July. | Morn. | Noon. | Even. | July. | Morn. | Noon. | Even. | July. | Morn. | Noon. | Even. | July. | Morn. | Noon. | Even. |
| 1 | 54 | 69 | 58 | | | | 1 | 68 | 80 | 77 | 1 | 53 | 83 | 68 | 1 | 54 | 86 | 70 | | | | |
| 2 | 53 | 69 | 57 | | | | 2 | 69 | 84 | 78 | 2 | 62 | 84 | 72 | 2 | 59 | 87 | 73 | | | | |
| 3 | 55 | 70 | 57 | | | | 3 | 69 | 83 | 77 | 3 | 62 | 88 | 72 | 3 | 58 | 92 | 72 | | | | |
| 4 | 55 | 70 | 56 | | | | 4 | 70 | 90 | 82 | 4 | 64 | 92 | 82 | 4 | 60 | 96 | 84 | | | | |
| 5 | 56 | 70 | 55 | | | | 5 | 78 | 92 | 84 | 5 | 68 | 96 | 84 | 5 | 62 | 107 | 87 | | | | |
| 6 | 58 | 68 | 54 | | | | 6 | 79 | 94 | 83 | 6 | 70 | 103 | 94 | 6 | 63 | 110 | 97 | | | | |
| 7 | 54 | 67 | 55 | | | | 7 | 80 | 94 | 82 | 7 | 74 | 102 | 90 | 7 | 65 | 110 | 93 | | | | |
| 8 | 60 | 69 | 56 | | | | 8 | 81 | 95 | 83 | 8 | 72 | 100 | 88 | 8 | 58 | 108 | 90 | | | | |
| 9 | 56 | 67 | 58 | | | | 9 | 81 | 95 | 83 | 9 | 70 | 100 | 88 | 9 | 57 | 107 | 91 | | | | |
| 10 | 55 | 68 | 56 | | | | 10 | 81 | 94 | 83 | 10 | 70 | 101 | 87 | 10 | 56 | 108 | 85 | | | | |
| 11 | 54 | 69 | 57 | | | | 11 | 79 | 93 | 82 | 11 | 68 | 96 | 84 | 11 | 53 | 105 | 86 | | | | |
| 12 | 56 | 70 | 57 | | | | 12 | 78 | 94 | 84 | 12 | 68 | 95 | 82 | 12 | 58 | 105 | 90 | 12 | 76 | 93 | 82 |
| 13 | 54 | 68 | 52 | | | | 13 | 77 | 92 | 81 | 13 | 69 | 98 | 83 | 13 | 59 | 107 | 92 | 13 | 78 | 93 | 80 |
| 14 | 55 | 66 | 54 | | | | 14 | 79 | 91 | 81 | 14 | 68 | 98 | 88 | 14 | 59 | 106 | 93 | 14 | 80 | 94 | 82 |
| 15 | 54 | 66 | 53 | 29.82 | 29.82 | 29.81 | 15 | 77 | 90 | 81 | 15 | 70 | 96 | 83 | 15 | 59 | 107 | 95 | 15 | 78 | 92 | 82 |
| 16 | 56 | 72 | 59 | 29.79 | 29.80 | 29.80 | 16 | 76 | 90 | 80 | 16 | 62 | 90 | 76 | 16 | 54 | 102 | 94 | 16 | 77 | 91 | 80 |
| 17 | 56 | 70 | 57 | 29.82 | 29.83 | 29.80 | 17 | 77 | 93 | 82 | 17 | 62 | 88 | 80 | 17 | 53 | 100 | 94 | 17 | 78 | 94 | 83 |
| 18 | 55 | 70 | 60 | 29.82 | 29.82 | 29.79 | 18 | 78 | 93 | 81 | 18 | 62 | 88 | 82 | 18 | 53 | 100 | 92 | 18 | 78 | 95 | 82 |
| 19 | 58 | 74 | 61 | 29.72 | 29.78 | 29.76 | 19 | 79 | 92 | 83 | 19 | 62 | 88 | 84 | 19 | 53 | 99 | 92 | | | | |
| 20 | 57 | 75 | 59 | 29.73 | 29.73 | 29.71 | 20 | 84 | 90 | 82 | 20 | 65 | 98 | 86 | 20 | 55 | 100 | 88 | | | | |
| 21 | 54 | 67 | 55 | 29.73 | 29.73 | 29.72 | 21 | 85 | 90 | 81 | 21 | 70 | 102 | 90 | 21 | 58 | 107 | 89 | | | | |
| 22 | 56 | 68 | 54 | | | | 22 | 85 | 91 | 83 | 22 | 70 | 106 | 92 | 22 | 57 | 112 | 94 | | | | |
| 23 | 55 | 68 | 55 | | | | 23 | 86 | 94 | 82 | 23 | 70 | 106 | 94 | 23 | 59 | 113 | 93 | | | | |
| 24 | 55 | 67 | 53 | | | | 24 | 80 | 92 | 81 | 24 | 72 | 105 | 95 | 24 | 60 | 113 | 94 | | | | |
| 25 | 54 | 75 | 57 | 29.72 | 29.80 | 29.73 | 25 | 73 | 90 | 86 | 25 | 72 | 105 | 96 | 25 | 61 | 105 | 92 | | | | |
| 26 | 54 | 78 | 55 | 29.75 | 29.76 | 29.76 | 26 | 72 | 92 | 86 | 26 | 72 | 109 | 96 | 26 | 64 | 122 | 105 | | | | |
| 27 | 52 | 75 | 55 | 29.72 | 29.78 | 29.80 | 27 | 75 | 100 | 85 | 27 | 66 | 101 | 85 | 27 | 61 | 109 | 101 | | | | |
| 28 | 53 | 78 | 54 | 29.80 | 29.83 | 29.82 | 28 | 77 | 99 | 85 | 28 | 68 | 100 | 90 | 28 | 62 | 107 | 98 | | | | |
| 29 | 53 | 62 | 56 | 29.79 | 29.79 | 29.74 | 29 | 79 | 109 | 89 | 29 | 70 | 110 | 98 | 29 | 62 | 107 | 97 | | | | |
| 30 | 65 | 78 | 55 | 29.76 | 29.74 | 29.73 | 30 | 80 | 101 | 83 | 30 | 80 | 105 | 92 | 30 | 63 | 104 | 94 | | | | |
| 31 | 52 | 67 | 55 | 29.74 | 29.75 | 29.72 | 31 | 72 | 99 | 81 | 31 | 70 | 97 | 88 | 31 | 57 | 100 | 90 | | | | |

At San Francisco, in July, the mornings were over-cast and foggy. At 9, A. M. till 2, P. M., warm and clear. At 2, P. M., the sea breeze sets in strong, filling the air with sand and dust. The winters, at San Francisco, are milder than summer. The winds coming from the south in winter and from the west in summer.

In the valleys there is a gentle breeze in the afternoon. No clouds, or fogs, or rain are known from April till December. Most of the rainy season occurs in January, February, and March.

Sacramento is about 65 miles (air line) from San Francisco, N. E. Marysville, 50 miles N. of Sacramento. Mormon Island in the Mining Region (mountainous) on the South Fork of the American River, 35 miles N. E. from Sacramento. You will notice a great variation in the temperature of San Francisco and the other towns. San Francisco is outside of the Coast Range of Mountains. Sacramento and Marysville are located in Sacramento valley (inside the Coast Range,) Stockton in San Joaquin valley, Mormon Island, Mountain Region. In passing up or down the Sacramento River in a distance of 3 miles (the gap of the Coast Range) the Thermometer changes from 15° to 20°. At Benecia and San Jose, the Thermometer varies but little in summer and winter from 75°.

METEOROLOGICAL TABLE,

| | Thermometer. | | | Barometer. | | |
|---|---|---|---|---|---|---|
| 1852. Sept. | Morn. | Noon. | Night. | Morn. | Noon. | Night. |
| 5 | 54 | 70 | 56 | 29.75 | 29.78 | 29.75 |
| 6 | 54 | 68 | 54 | 29.78 | 29.80 | 29.80 |
| 7 | 51 | 73 | 54 | 29.80 | 29.80 | 29.79 |
| 8 | 52 | 78 | 55 | 29.79 | 29.80 | 29.77 |
| 9 | 53 | 85 | 62 | 29.79 | 29.82 | 29.80 |
| 10 | 60 | 97 | 76 | 29.80 | 29.79 | 29.79 |
| 11 | 66 | 98 | 61 | 29.78 | 29.75 | 29.70 |

Wind chiefly westerly.

Sunday, 5th. Cloudy and misty; partly clear; moderate wind. 6th. Clear; moderate wind. 7th and 8th. Clear and pleasant breeze. 9th. Clear and warm; light breeze. 10th. Clear and hot; nearly calm. 11th. Clear and hot; light breeze; cool and windy in the evening.

This week will long be remembered for the extraordinary heat of the last two days. On the 9th the mercury rose to 85°, which was one degree above any former observation on my journal, extending back two years. On the 10th it reached 97°, and on the 11th, 98°. The night of the 10th was so warm that persons slept with open windows, in violation of all precedent. Toward sunset on the 11th, a sea breeze sprang up and brought down the temperature from 90° at 5 o'clock to 75° at 6, a change of 15 degrees in one hour. By 10 o'clock it was 61, making a descent of 29 degrees in 5 hours. The change from 3, P. M., on the 11th, to 5, A. M., on the 12th, a period of 14 hours, was 45 degrees. The following statement shows the temperature for each hour of the two days:

| Hour. | 10th. | 11th. | Hour. | 10th. | 11th. |
|---|---|---|---|---|---|
| 6, A. M. | 60° | 66° | 3, P. M. | 96° | 98° |
| 7, " | 66 | 70 | 4, " | 93 | 94 |
| 8, " | 71 | 84 | 5, " | 91 | 90 |
| 9, " | 74 | 88 | 6, " | 85 | 80 |
| 10, " | 78 | 82 | 7, " | 88 | 68 |
| 11, " | 83 | 86 | 8, " | 80 | 66 |
| 12, " | 90 | 91 | 9, " | 78 | 64 |
| 1, P. M. | 96 | 97 | 10, " | 76 | 61 |
| 1, " | 97 | 98 | | | |

The thermometer hanging in front of my office facing the north, entirely shaded from the direct rays of the sun, but exposed to some reflection, stood at 100° on the 10th, and 102° on the 11th. By placing several instruments in different situations where the air circulated freely and where there was the least reflection, I obtained the foregoing tables. In the newspaper notices of the weather throughout the State, the observations are generally made without this caution, and hence in comparing the recent hot weather in this city with the climate in the interior as represented in the papers, it is proper to deduct from two to five degrees from the observations in the interior, or otherwise to place the extreme temperature of the 10th and 11th inst. in San Francisco at 100° and 102°. It may be well to add that the air was loaded with smoke, leading some to infer that the heat proceeded from the fires on the plains and hills, while others who were looking to another source for this unusual temperature, perceived the odor of brimstone! There is no occasion to seek the cause of heat beyond the absence of the sea breeze. If this much vilified agitator should abandon the field, the citizens of San Francisco would be broiled every day during the summer, without the aid of fire and brimstone.

San Francisco, Sept., 1852.

